# Challenging the No-Stent Zone: Intravascular Lithotripsy for Common Femoral Artery Disease

**DOI:** 10.3390/jcm14186492

**Published:** 2025-09-15

**Authors:** Ivan B. Ye, Stefanos Giannopoulos, Angela A. Kokkosis, Patrick T. Jasinski, Nicos Labropoulos

**Affiliations:** 1 NYU Langone Health Long Island, Division of Vascular Surgery, Department of Surgery, Garden City, NY 11530, USA; ivan.ye@nyulangone.org (I.B.Y.); angela.kokkosis@stonybrookmedicine.edu (A.A.K.); 2 Stony Brook Medicine, Division of Vascular and Endovascular Surgery, Department of Surgery, Stony Brook University Hospital, Stony Brook, NY 11794, USA; stefanos.giannopoulos@stonybrookmedicine.edu (S.G.); nicos.labropoulos@stonybrookmedicine.edu (N.L.)

**Keywords:** IVL, shockwave, CFA, calcification, PAD, peripheral artery disease

## Abstract

**Background/Objectives:** Calcified atherosclerotic disease of the common femoral artery (CFA) is a unique therapeutic challenge. Although endarterectomy has been the gold standard for CFA disease treatment, the evolution of endovascular techniques has led to increasing interest in minimally invasive alternatives to open surgery. Intravascular lithotripsy (IVL) offers calcium modification, facilitating vessel compliance and enhancing the effect of adjunctive therapies. **Methods:** A systematic review of current literature was conducted to summarize data on clinical outcomes and perioperative complications of IVL for the treatment of calcified CFA disease. Eligible studies up to July 2025 were included. Demographics, procedural and follow-up data were extracted and analyzed. **Results:** A total of 304 patients across 9 studies were included. The majority of the patients were males (68%) with hypertension (88%), dyslipidemia (75%) and smoking history (65%). Of the included patients, 56% had chronic limb-threatening ischemia (CLTI), with a previous intervention reported in about one-third of them. Additional calcium-modifying technology was used at the discretion of the operator in 11% of cases. The overall dissection rate was 2% (95% confidence interval (CI): 0–6%) with only five cases having flow-limiting dissections. Stenting rate was 3% (95% CI: 0–11%). No perforations or distal embolization events were reported by the rest of the studies apart from one study that had one such case. The overall periprocedural complication rate was 5% (95% CI: 0–13%)**.** The 12-month target lesion revascularization (TLR) rate was 9% (95% CI: 3–18%). **Conclusions:** IVL could be a safe alternative endovascular option to open repair for treating calcified CFA disease with low rates of procedural complications. Further studies are needed to clarify IVL’s role in clinical practice, particularly for patients who are poor candidates for open surgery.

## 1. Introduction

Atherosclerotic disease of the common femoral artery (CFA) is a unique therapeutic challenge due to the anatomic location, frequent involvement of bifurcation points and dynamic mechanical stressors. The gold standard for the treatment of atherosclerotic disease in this area is femoral endarterectomy, offering durable patency and clinical efficacy. However, it is associated with perioperative morbidity in patients with significant comorbidities or challenging anatomy (e.g., redo operations, severely calcified vessels, need for bypass procedure) [1]. The evolution of endovascular technology and techniques has led to increasing interest in minimally invasive alternatives to open surgery for CFA lesions. However, conventional balloon angioplasty and stenting have been met with limited enthusiasm in this area due to concerns about embolization, dissection, lack of endovascular bail-out, stent fracture, restenosis and impaired access for future interventions [2,3].

Intravascular lithotripsy (IVL), a novel endovascular modality that delivers acoustic shockwaves to disrupt calcified plaque, has been successfully used for peripheral arterial calcified lesions. By modifying both intimal and medial calcium while preserving the underlying arterial architecture, IVL facilitates luminal gain at lower angioplasty pressures with reduced barotrauma and dissection risk compared to traditional balloon angioplasty [4]. Recent observational studies and registry data suggest that IVL may offer promising technical success and favorable short-term outcomes in the treatment of CFA atherosclerosis, even in complex calcified disease [5]. Nonetheless, high-level evidence is limited, and there continues to be no consensus regarding its efficacy and safety at this particular vascular bed.

The purpose of this study was to conduct a systematic review of the current literature and summarize pooled data on clinical outcomes and perioperative complications of IVL for the treatment of atherosclerotic disease in the CFA. This could support evidence-based decision-making, patient selection and inform consent discussions, in cases where open CFA disease repair is not feasible due to lesion or patient characteristics and an endovascular option is the only option.

## 2. Materials and Methods

This article was prepared according to the “Preferred Reporting Items for Systematic Reviews and Meta-analyses” (PRISMA) guidelines [6]. An English-language literature review was carried out up to June 2025 to investigate the clinical outcomes of IVL for CFA disease.

### 2.1. Search Strategy

Systematic searches were conducted in PubMed/Medline, Scopus and Cochrane Central databases. The search was performed by two independent investigators, who were blind to each other. Any disagreements or discrepancies were resolved by consensus and in agreement with a third independent investigator. No restrictions were imposed on gender or geography. The electronic search was supplemented by a manual search of the reference lists of eligible articles.

### 2.2. Eligibility Criteria

A study was included in this systematic review if it fulfilled the following predefined criteria: (i) prospective or retrospective observational analyses reporting on patients who underwent IVL for CFA disease, (ii) studies that included only subjects with calcified CFA disease, and (iii) studies published up to July 2025; additionally, (iv) single-arm studies or double-arm studies were included as well if information was provided separately for the CFA disease cases. Published abstracts and case reports were not included. When duplicates were identified, the study with the most complete dataset was utilized. Also, care was taken to not include studies with overlapping population in the same analysis. This systematic review utilized study-level data. No institutional review board approval was required.

### 2.3. Data Extraction

The data extraction process involved two reviewers who independently extracted the relevant data from the eligible studies and were blind to each other; final decision was reached by consensus. Data from eligible studies were abstracted into an Excel spreadsheet (Microsoft Corp, Redmond, Wash). The primary endpoints were perioperative complications, including flow-limiting and non-flow-limiting dissection, bail-out stent placement, perforation and distal embolization. Secondary endpoints included major amputation, all-cause mortality and target lesion revascularization during follow-up. Additional data extracted from each study included demographic information (mean age of study population and gender), medical comorbidities, lesion characteristics (chronic total occlusion, calcification, lesion length) and procedural details (shockwave pulses, drug-coated balloon (DCB) angioplasty, and adjunctive calcium-modifying technology).

### 2.4. Study Risk of Bias Assessment

The risk of bias for the articles included was evaluated by two investigators applying the Risk of Bias in Non-Randomized Studies–of Interventions (ROBINS-I) tool [7].

### 2.5. Statistical Analysis

The cumulative incidence of primary and secondary endpoints was estimated and synthesized with the corresponding 95% confidence intervals (CIs). The CIs were based on score (Wilson) and exact binomial (Clopper–Pearson) procedures [8]. A test of whether the summary effect measure was equal to zero was given, as well as a test for heterogeneity, i.e., investigating whether the true effect in all studies was the same. Heterogeneity was quantified using the I-squared measure [9]. Forest plots were used to graphically display the effect size (ES) in each study and the pooled estimates, which in this study was the proportion of each outcome. The methodologies of Hozo et al. and the Wan et al. were applied to estimate the means and standard deviations of continuous variables whenever medians were reported [10,11]. All statistical analyses were carried out using STATA 14.1 (StataCorp, College Station, TX, USA).

## 3. Results

### 3.1. Literature Search

**Figure 1:**
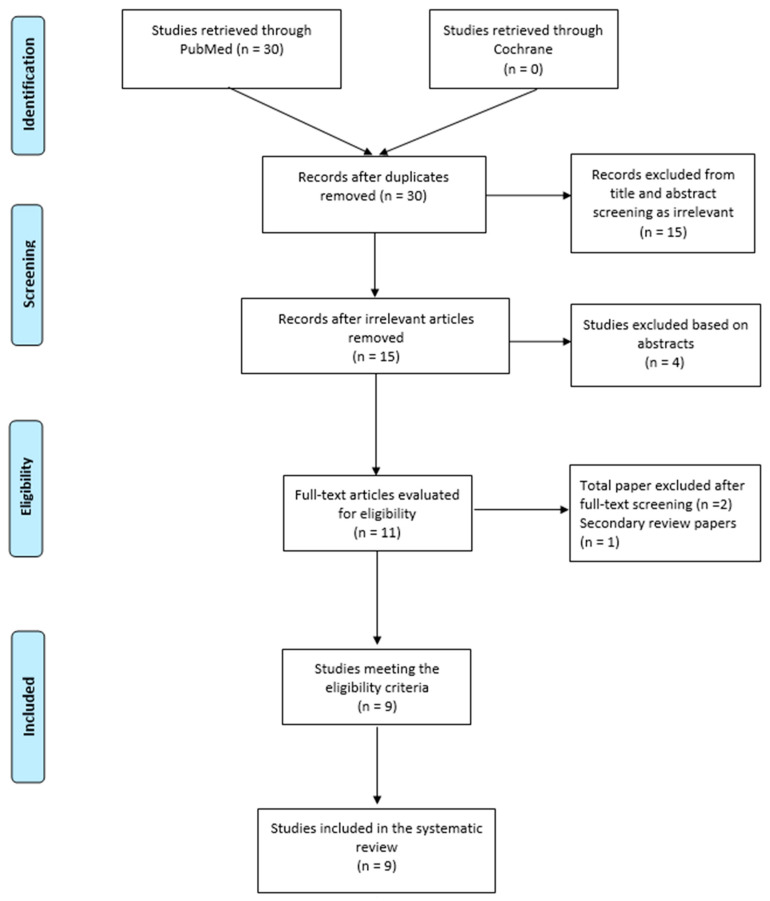
PRISMA search flow diagram (last search 2025).

The systematic search yielded 30 eligible journal articles. After reviewing abstracts and titles, 15 studies were identified to be eligible for this systematic review [4,5,12,13,14,15,16,17,18]. After full-text evaluation, eleven studies deemed to be relevant, out of which two studies were excluded—one study was a secondary review article, and the other one did not report separate outcomes for CFA cases. The PRISMA search flow diagram is illustrated in Figure 1.

### 3.2. Study Characteristics

The nine included studies originated from the United States, Spain, Germany, and Italy. Among the included studies, seven were retrospective single-center observational studies [12,13,14,15,16,17,18], while two studies were prospective multi-center observational studies [4,5]. Treatment modality varied between studies, although in all cases IVL technology was used. In one study, only IVL was used [12], while in three studies, IVL and DCB were performed [13,14,17]. In the remaining five studies, IVL was used with/without adjunctive therapy and/or additional DCB or plain balloon angioplasty [4,5,15,16,18]. Details regarding the treatment modalities utilized in each study are summarized in Table 1. Two studies shared a subset of patients [16,17], with the more comprehensive dataset used for primary analysis [16]. Core lab adjudication was utilized in two studies [4,5]. The risk of bias across the studies is outlined in Supplementary Table S1.

### 3.3. Population Characteristics

A total of 304 patients and 310 lesions were included. Mean age ranged from 72 to 75 years of age. In a pooled analysis of baseline demographics, majority of patients were male (68%; 95% CI: 55–80%) with comorbidities including hypertension (88%; 95% CI: 75–97%), dyslipidemia (75%; 95% CI: 57–90%), and history of smoking (65%, 95% CI: 37–88%). Meanwhile, diabetes (37%; 95% CI: 25–51%), heart disease (40%; 95% CI: 21–60%), chronic kidney disease (21%; 95% CI: 10–34%), and cerebrovascular disease (18%; 95% CI: 11–27%) were less common. Baseline population characteristics and comorbidities are summarized in Table 1. All patients included in the analysis had calcified CFA disease. Three studies reported a mean ankle-brachial index (ABI) ranging from 0.67 to 0.7. A total of 56% of the patients had critical limb-threatening ischemia (CLTI) at baseline (95% CI: 31–81%). Prior intervention for peripheral artery disease (PAD) was noted in 30% of patients (95% CI: 16–45%).

### 3.4. Lesion Characteristics

Important lesion and procedural characteristics are summarized in Table 2. Overall, about one-third of the cases had occlusive CFA disease (30%; 95% CI: 13–50%), and moderate or severe calcification was observed in 94% of the lesions (95% CI: 86–99%). Among the studies with available data, severe calcification was reported in 60% (95% CI: 33–85%). Lesion lengths and calcification lengths ranged from 26 to 56 mm and 28 to 77 mm, respectively, while mean stenosis ranged from 72.3% to 86.7% among studies with available data. Mean shockwave pulses ranged from 140 to 218. Post-IVL residual stenosis ranged from 25.6% to 40%. Drug-coated balloon (DCB) angioplasty was used in 90% (95% CI: 75–99%) of the lesions. Pre-dilation, stenting and utilization of drug-eluting technology was at the discretion of the operator. The utilization of adjunctive calcium-modifying technology including specialty balloons and/or atherectomy was reported by all included studies. Overall, additional calcium-modifying technology was used in 11% of the lesions (95% CI: 0–30%) at the discretion of the operator.

### 3.5. Perioperative Outcomes

**Figure 2:**
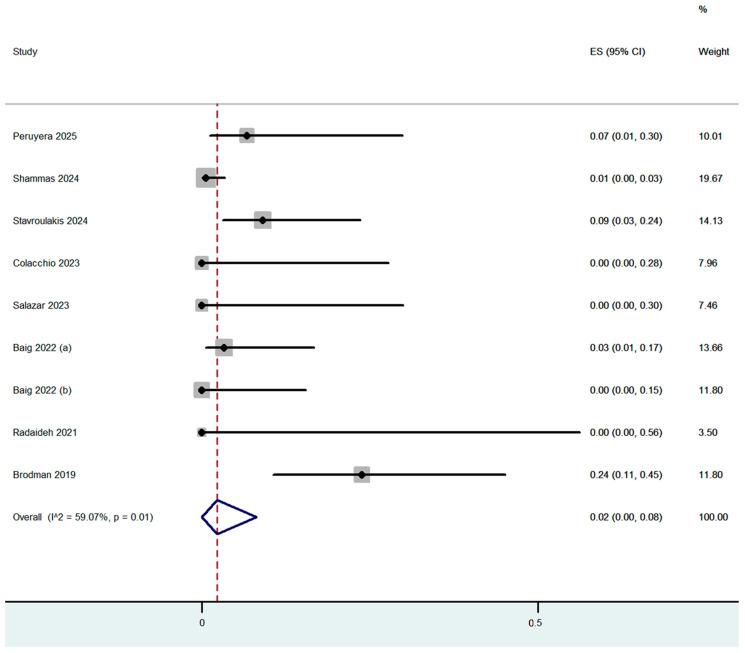
Proportion estimate of any dissections (effect size, ES) [5,12,13,14,15,16,17,18,19].

**Figure 3:**
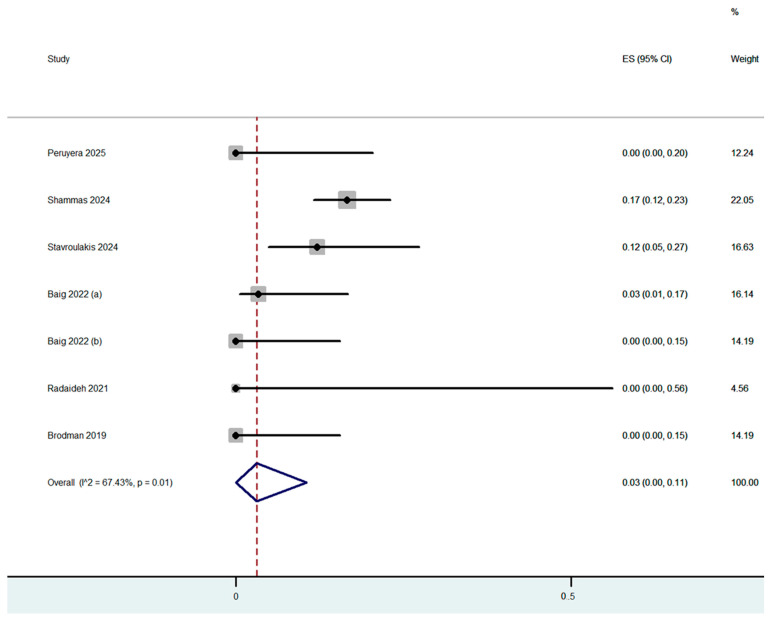
Proportion estimate of bail-out stenting (effect size, ES) [5,12,13,16,17,18,19].

**Figure 4:**
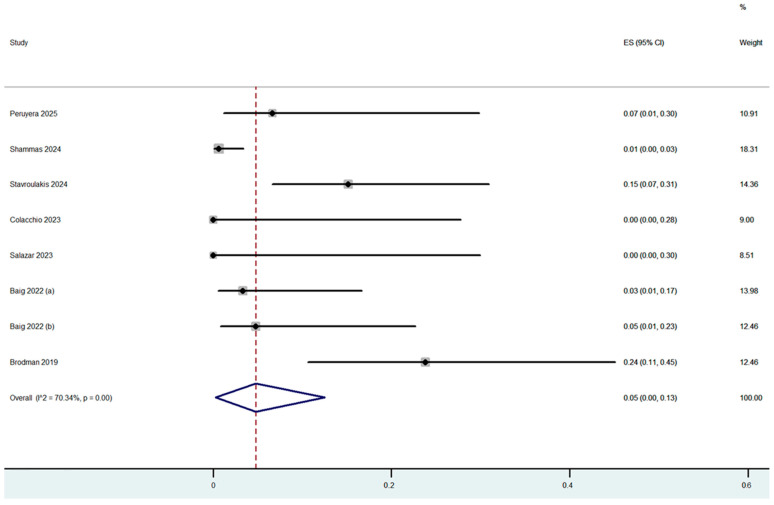
Proportion estimate of any procedural complications (effect size, ES) [5,12,13,14,15,16,17,19].

Procedure complications were reported in all nine studies (Table 3). The overall dissection rate was 2% (95% CI: 0–6%) (Figure 2) with only 5 cases of flow-limiting dissections out of the 250 cases which were treated (4 with stenting). Flow-limiting dissections were observed in only three out of the nine included studies. Bail-out stenting was necessary for optimal angiographic result in 3% (95% CI: 0–11%) of the cases, with the highest rate being reported in the study by Shammas et al., in which adjunctive calcium-modified technologies were used (Figure 3). Bail-out stenting was necessary in cases of three out of the nine included studies. One case of distal embolization and perforation was reported by Stavroulakis et al. However, the perforation was at superficial femoral artery (SFA) and was treated with the placement of a covered stent. No CFA perforations or distal embolization events were reported by the rest of the studies. The overall combined periprocedural complication rate was 5% (95% CI: 0–13%) (Figure 4).

### 3.6. Mid-Term Outcomes

**Figure 5:**
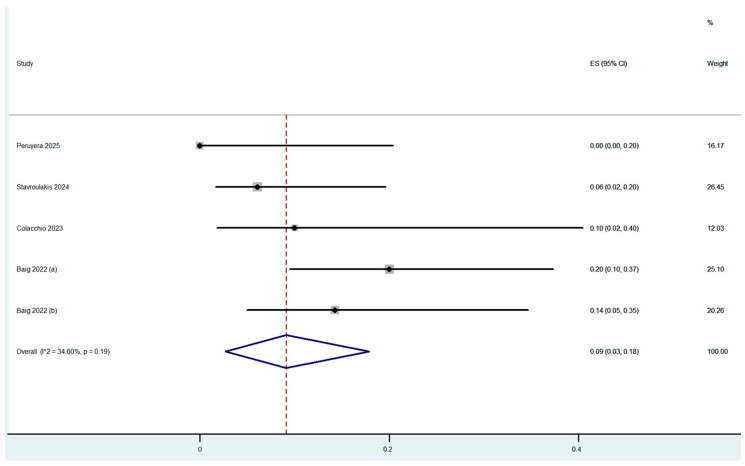
Proportion estimate of 12-month TLR rate (effect size, ES) [12,13,14,16,17].

Overall, five studies, including 109 patients, provided mid-term data. Mean follow-up ranged from 11 to 18 months, with an estimated mean study follow-up of 14.9 (standard deviation (SD): 2.7) months. The pooled major amputation rate during follow-up was 2% (95% CI: 0–14%). All-cause mortality was 8% (95% CI: 1–21%). The 12-month target lesion revascularization (TLR) rate was 9% (95% CI: 3–18%) (Figure 5). Primary patency data were provided in the study by Stavroulakis et al., with a reported rate of 72% during a mean follow up of 13.5 months. Long-term outcomes and follow-up are summarized in Supplementary Table S2.

## 4. Discussion

This systematic review of nine recent observational studies involving 310 CFA lesions demonstrated that IVL is safe and effective in treating complex CFA lesions, with the majority of lesions having severe calcification and lesion lengths exceeding 50 mm. Short-term outcomes showed an overall low complication rate, with low dissection (2%) and bail-out stenting rate (3%), with only one case of distal embolization. Although long-term outcomes are limited, the 12-month follow-up revealed a 9% TLR rate and 2% major amputation rate, suggesting favorable sustained outcomes after IVL. In general, the Shockwave^®^ peripheral IVL system has integrated lithotripsy emitters and is designed to enhance percutaneous transluminal balloon angioplasty (PTA) at low balloon pressures. After a low-pressure balloon dilation of up to 4 atm, mechanical pulse waves are produced that alter the structure of the stenosed vessel [20,21].

The efficacy of IVL for treating calcified femoropopliteal lesions has been established in the Disrupt PAD I, II and III studies [19,22,23]. Disrupt PAD I was the first to demonstrate the safety of IVL in 35 patients with calcified lesions [22]. Disrupt PAD II confirmed these findings with successful treatment using IVL in a cohort of 60 patients, reporting a final residual stenosis of 24.2% [19]. Disrupt PAD III, a randomized control trial of 306 patients, compared IVL to conventional angioplasty and showed higher procedural success and a greater proportion of lesions with residual stenosis <30% (66.4% vs. 51.9%) [23,24]. The acute outcomes of Shockwave^®^ balloon were excellent; however, re-stenosis was prevalent, given the lesion complexity, and frequent additional modalities, such as drug-eluting technology, are necessary to improve long-term patency.

While the Disrupt PAD studies demonstrated the efficacy of IVL in femoropopliteal lesions, CFA lesions were excluded. The Disrupt PAD III Observational Study was a prospective registry evaluating IVL use for calcified PAD in routine clinical practice [25]. Among 1373 patients, target lesions included femoropopliteal (61%), iliac (15.8%), common femoral (10.7%) and infrapopliteal arteries (12.8%). A subset analysis by Shammas et al. examined 163 patients with CFA lesions from this registry, which was included in this study [5]. The study by Shammas et al. showed that no vascular complications (flow-limiting dissections, perforations, embolization, slow or no reflow, or abrupt closure) were present at the end of the procedure, with only one (0.8%) flow-limiting dissection immediately following IVL treatment, highlighting the safety of IVL for this vascular bed, although stenting rate was 16.5% [5].

Femoral endarterectomy remains the gold standard treatment for CFA atherosclerotic disease, especially for moderate/severe calcification cases, given limited endovascular options in this “no-stent zone.” PTA is associated with acute dissection in 47% to 88% of cases overall [26,27], and vessel calcification can increases the risk of flow-limiting dissection even more [28,29], which could eventually lead to higher bail-out stenting rates. However, stenting in this region risks compromising profunda femoris outflow, is subject to mechanical stress from hip movement, and may hinder future surgical or endovascular access. Additionally, appropriate sizing of balloons and/or stents may often be challenging due to calcification, and more advanced imaging with intravascular ultrasound (IVUS) may be necessary to better assess the plaque volume and composition [30]. Interestingly, the degree of calcification has been directly correlated with higher rates of TLR for endovascular infrainguinal procedures [31]. However, this may not be routinely available in clinical practice. IVL has emerged as a novel calcium-modifying technique and may be a feasible endovascular option for patients with complex CFA disease, reducing the risk of post-balloon angioplasty dissections and, as such, minimizing the need for stenting in order to achieve acceptable angiographic outcome.

Additionally, common femoral endarterectomy has been associated with substantial surgical morbidity, including an 8% incidence of wound complications and a 15% rate of 30-day mortality/morbidity, with risks amplified in high-risk patients [32]. IVL offers a less-invasive alternative for treating CFA lesions, potentially reducing the complication rate; however, there was no study published summarizing all the current literature regarding the perioperative risks of IVL. In this systematic review, the pooled procedural complication rate was 5%, with flow-limiting dissections reported only in 5 out of 250 cases and 3% overall stenting rate. Meanwhile, no perforations were noted in the CFA region and only one distal embolization was reported. These results aligned with the Disrupt PAD III study, which reported lower rates of flow-limiting dissections (1.4% vs. 6.8%) and bail-out stenting (4.6% vs. 18.3%) in the IVL cohort compared to the angioplasty cohort when utilized for infrainguinal disease [24]. Overall, IVL appears to have acceptable risks when utilized at the CFA segment and should be strongly considered when open repair is not feasible due to patient (e.g., high risk for wound complications in obese populations or patients with previous groin procedures, multiple comorbidities, limited life expectancy, etc.) or lesion characteristics (e.g., very long segment, severe calcification, not “good” proximal and distal clamping sites, etc.).

This study has several limitations. First, although the evidence supporting IVL for CFA disease is promising, it remains limited by a lack of high-quality studies. All included studies were observational and predominantly retrospective and single-center, introducing potential selection and reporting bias. Only two studies employed independent core laboratory adjudication for angiographic outcomes. Technical and procedural success was poorly defined among the studies. Furthermore, clinical outcomes were inconsistently reported with limited data on symptom-based endpoints, such as wound healing and symptom resolution.

Procedural techniques varied across studies, including differences in the use of pre- and post-IVL dilation, drug-coated balloons and adjunctive atherectomy. As no patient-level data were available, and outcomes were not consistently reported separately for cases in which IVL was used as a standalone treatment, it was not possible to perform a sensitivity analysis based on treatment strategies. While the potential effect of adjunctive modalities (e.g., DCB, atherectomy) should be considered when interpreting the results of the current study, it is important to emphasize that this analysis still demonstrates that IVL—with or without adjunctive therapies—was associated with a very low complication rate when utilized for this “no-stent” zone. Another important limitation to highlight is that IVL is most effective in cases of circumferential calcification [33]. CFA plaque is usually eccentric and posterior and therefore the ability of IVL to achieve complete calcium modification at that area may be limited. Careful assessment of the plaque morphology is essential to identify patients that would benefit the most from IVL use, although the overall complication rate was very low. Given these limitations, further prospective studies with standardized IVL protocols and outcome definitions are needed to better determine the efficacy of IVL at that particular vascular bed. 

## 5. Conclusions

This study summarized all current literature regarding IVL use for calcified CFA disease and provided pooled estimates of periprocedural complication rates. More specifically, the study demonstrated that IVL could be a safe and effective alternative endovascular option for patients who are not candidates for surgical repair, with low rates of adverse events. Although long-term data remain limited, reported TLR rate during a mean follow-up of 15 months was promising. Further studies are needed to clarify IVL’s role for “no stent” zones in clinical practice, particularly for patients who are poor candidates for open repair.

## Figures and Tables

**Table 1:** Baseline population characteristics.

Study	Total (N)	Treatment Modality	Age (Years), Mean (SD)	Males (%)	Dyslipidemia (%)	Diabetes (%)	HTN (%)	Heart Disease (%)	CVD (%)	CKD (%)	Smoking History (%)	Prior Intervention (%)	Baseline ABI, Mean (SD)	CLTI (%)
Peruyera 2025 [12]	15	IVL only	72.5 (9.7)	86.7	73.3	60	73.3	33.3	20	6.7	73.3	13.3		100
Shammas 2024 [5]	163	IVL + adjunctive therapy	72.1 (8.6)	76	88.3	37.4	93.3	23.9	12.9	20.9	88.3		0.7 (0.3)	26.9
Stavroulakis 2024 [13]	33	IVL + DCB	73 (10)	51.5	60.6	33.3	93.9	48.5	18.2	51.5	36.4	33.3	0.67 (0.39)	42.4
Colacchio 2023 [14]	20	IVL + DCB	75 (9.2)	35	45	10	50	25		15	25	40		25
Salazar 2023 [15]	9	IVL +/− adjunctive therapy (no atherectomy)					100							100
Baig 2022 (b) [17]	50	IVL +/− atherectomy +/− DCB	74.6 (8.0)	74	92	50	94	68	28	14	88		0.67 (0.2)	30
Radaideh 2021 [18]	3	IVL + atherectomy												
Brodman 2019 [19]	21	IVL + PTA/DCB (x1 atherectomy)	71.9 (10.1)	76.2										

N: number; yrs: years; HTN: hypertension; CVD: cerebrovascular disease; CKD: chronic kidney disease; ABI: ankle-brachial index; CLTI: chronic limb-threatening ischemia; IVL: intravascular lithotripsy; DCB: drug-coated balloon angioplasty; PTA: percutaneous transluminal balloon angioplasty.

**Table 2:** Lesion and procedural characteristics.

Study	Patients; Lesions (N)	CTO (%)	Moderate/Severe Calcification (%)	Severe Calcification (%)	Lesion Length (mm), Mean (SD)	Calcification Length (mm), Mean (SD)	Diameter Stenosis (%), Mean (SD/Range)	Reference Vessel Diameter (mm), Mean (SD)	Pre-Dilation (%)	Mean Number of Shockwave Pulses (SD)	Post-IVL Diameter (%), Mean (SD/Range)	Adjunctive Calcium-Modifying Technology (%)	DCB (%)
Peruyera 2025 [12]	15; 15	53.3	100	66.7			86.74 (16.22)	6.26 (0.71)	20			0	93.3
Shammas 2024 [5]	163	18.4	95.1		53.6 (53.1)	77.4 (61.5)	74.8 (17.7)	6.3 (1.1)	28	188.5 (102.6)	29.2 (16.5)	35.9	68.9
Stavroulakis 2024 [13]	33	12.1			56 (20.7)							0	100
Colacchio 2023 [14]	10; 12	100	83.3	66.7	28.2 (10.7)	28.17 (10.74)	80 (78.7–90)				40 (31.2–40)	0	
Salazar 2023 [15]	9						82.6 (20.6)				25.6 (26.6)	0	
Baig 2022 (b) [17]	50; 54	11.1	87	62.9	25.9 (10.3) out of 30 cases		76.6 (13.0)	6.80 (1.54)	17 cases of pre or post-dilation	218 (105)		38.9	83.3
Radaideh 2021 [18]	3		100	100								100	100
Brodman 2019 [19]	21		100	71.4	37.8 (16.7)	61.6 (30.7)	72.3 (12.8)	6.1 (0.8)	0	140 (58)		4.8	85.7

N: number; CTO: chronic total occlusion; SD: standard deviation; IVL: intravascular lithotripsy; DCB: drug-coated balloon.

**Table 3:** Perioperative outcomes.

Study	Total (N)	Flow-Limiting Dissection (%)	Non-Flow-Limiting Dissection (%)	Perforation (%)	Distal Embolization (%)	Bail-Out Stent (%)
Peruyera 2025 [12]	15	0	6.7	0	0	0
Shammas 2024 [5]	163	0.6	0	0	0	16.6
Stavroulakis 2024 [13]	33	6.1	3	3	3	12.1
Colacchio 2023 [14]	10	0	0	0	0	0
Salazar 2023 [15]	9	0	0	0	0	0
Baig 2022 (a) [16]	30	3.3	0	0	0	3.3
Baig 2022 (b) [17]	21	0	0	0	0	0
Radaideh 2021 [18]	3			0	0	0
Brodman 2019 [19]	21	0	23.8	0	0	0

N: number.

## Data Availability

The data presented in this study are available in publicly available published articles.

## Supplementary Materials

The following supporting information can be downloaded at https://www.mdpi.com/article/10.3390/jcm14186492/s1. Supplementary Table S1: Risk of Bias Assessment for Observational Studies (ROBINS-I Tool); Supplementary Table S2: Follow-up and Long-Term Outcomes.

## Abbreviations

CFA	Common femoral artery
SFA	Superficial femoral artery
IVL	Intravascular lithotripsy
DCB	Drug-coated balloon angioplasty
PTA	Percutaneous transluminal balloon angioplasty
CI	Confidence interval
ABI	Ankle-brachial index
CLTI	Critical limb-threatening ischemia
CTO	Chronic total occlusion
TLR	Target lesion revascularization
PAD	Peripheral artery disease
SD	Standard deviation
ES	Effect size
