# Supplementary material for: Challenging the No-Stent Zone: Intravascular Lithotripsy for Common Femoral Artery Disease
Source: J Clin Med. 2025 Sep 15;14(18):6492. doi: 10.3390/jcm14186492 (PMC12470913; doi:10.3390/jcm14186492)
Supplement: Supplementary file 1 [file jcm-14-06492-s001.zip › jcm-3861810-supplementary.pdf]

Supplementary Table S1 Risk of Bias Assessment for Observational Studies (ROBINS-I Tool)

Study	Confounding	Selection	Classification of Interventions	Deviations from Intended Interventions	Missing Data	Measurement of Outcomes	Selection of the Reported Result
Peruyera 2025	Moderate	Moderate	Moderate	Moderate	Moderate	Moderate	Moderate
Shammas 2024	Moderate	Low	Low	Moderate	Low	Low	Low
Stavroulakis 2024	Moderate	Moderate	Moderate	Moderate	Moderate	Moderate	Moderate
Colacchio 2023	Moderate	Moderate	Moderate	Moderate	Moderate	Moderate	Moderate
Salazar 2023	Moderate	Moderate	Moderate	Moderate	High	Moderate	Moderate
Baig 2022 (b)	Moderate	Moderate	Moderate	Moderate	Moderate	Moderate	Moderate
Radaideh 2021	Moderate	Moderate	Moderate	Moderate	High	Moderate	Moderate
Brodman 2019	Moderate	Moderate	Moderate	Moderate	High	Moderate	Moderate

Supplementary Table S2 Follow-up and Long-Term Outcomes

Study	Total (N)	Follow-up (Months), Mean (SD)	Major Amputations (N)	All-cause mortality (N)	TLR (N)
Peruyera 2025	15	14 (5.7)	1	3	0
Stavroulakis 2024	33	13.5 (8.6)	0	0	2
Colacchio 2023	10	11.1 (1.6)	1	1	1 (+2 TLR)
Baig 2022 (a)	30	Up to 18 months		6	6
Baig 2022 (b)	21	Up to 18 months		1	3

* SD: standard deviation, N: number of patients, TLR: target lesion revascularization
